# Autonomic Modulation and Symptomatic Efficacy of Transurethral Resection of the Prostate in Benign Prostatic Hyperplasia

**DOI:** 10.3390/life15101520

**Published:** 2025-09-26

**Authors:** Kuan-Yu Chen, Yun-Sheng Chen, Min-Hsin Yang, Yu-Hui Huang, Sung-Lang Chen

**Affiliations:** 1 Department of Urology, Chung Shan Medical University Hospital, Taichung 402, Taiwan; huracan1235@gmail.com (K.-Y.C.); barbarian06070136@gmail.com (M.-H.Y.); 2 Department of Obstetrics and Gynecology, Changhua Christian Hospital, Changhua 500, Taiwan; gracelucky028@gmail.com; 3 School of Medicine, Chung Shan Medical University, Taichung 402, Taiwan; 4 Department of Physical Medicine and Rehabilitation, Chung Shan Medical University Hospital, Taichung 402, Taiwan

**Keywords:** heart rate variability, lower urinary tract symptoms, transurethral resection of the prostate, benign prostatic hyperplasia

## Abstract

**Background**: Benign Prostatic Hyperplasia (BPH) causes Lower Urinary Tract Symptoms (LUTS), impairing quality of life (QoL). Transurethral Resection of the Prostate (TURP) is the gold-standard surgical treatment for Bladder Outlet Obstruction (BOO), but its effects on Autonomic Nervous System (ANS) function—assessed via Heart Rate Variability (HRV)—remains underexplored. To our knowledge, this is the first study to correlate HRV with specific LUTS domains pre- and post-TURP, establishing HRV as a potential biomarker for BPH management. **Methods**: In a prospective study, 242 men with BPH underwent TURP (2018–2024). Inclusion required age ≥ 50 years, International Prostate Symptom Score (IPSS) ≥ 8, and BOO evidence. HRV (Standard Deviation of Normal-to-Normal Intervals [SDNN], Low-Frequency/High-Frequency [LF/HF] ratio), IPSS, and QoL were assessed preoperatively and 3 months postoperatively. Paired *t*-tests, Pearson correlations, and multivariate regression (adjusted for age, Body Mass Index [BMI], prostate volume) were used (*p* < 0.05). **Results**: HRV (SDNN) increased from 36.97 ± 22.80 ms to 51.67 ± 27.59 ms (*p* = 0.032), and LF/HF ratio decreased from 1.63 ± 1.60 to 0.73 ± 0.52 (*p* = 0.028). IPSS fell from 18.5 ± 6.2 to 8.3 ± 4.1 (*p* < 0.001), with improved voiding (*p* = 0.004) and storage (*p* = 0.002) subscores. QoL improved from 3.5 ± 1.2 to 1.8 ± 0.9 (*p* = 0.003). HRV correlated inversely with IPSS voiding (r = −0.42, *p* = 0.012; r = −0.38, *p* = 0.019 post-TURP) and storage subscores (r = −0.29, *p* = 0.045). Older patients (≥65 years) and those with larger prostates (≥50 mL) showed greater improvements. **Conclusions**: TURP enhances LUTS, QoL, and ANS function. HRV’s correlation with LUTS suggests its biomarker potential, with possible cardiovascular benefits. Longitudinal studies are needed.

## 1. Introduction

Benign prostatic hyperplasia (BPH) is a common urological condition affecting aging males, characterized by nonmalignant enlargement of the prostate gland. This enlargement often leads to lower urinary tract symptoms (LUTS), including urinary frequency, urgency, nocturia, and weak stream, which significantly impair quality of life [1]. Transurethral resection of the prostate (TURP) remains the gold standard surgical treatment for moderate to severe BPH. It effectively relieves obstruction by removing excess prostatic tissue, resulting in substantial improvement in voiding function and symptom scores [2,3,4,5]. While TURP’s mechanical benefits are well documented, its influence on systemic physiological parameters, particularly autonomic nervous system (ANS) function, is less explored.

Recent research has begun to explore the role of ANS in voiding function [6,7,8]. McVary et al. [9] proposed that chronic bladder outlet obstruction may lead to sustained sympathetic activation, contributing to both voiding and storage symptoms. This hypothesis aligns with emerging evidence that autonomic modulation may influence detrusor muscle activity and bladder compliance.

Heart rate variability (HRV) is a sensitive marker of ANS activity, reflecting the balance between sympathetic and parasympathetic inputs [10,11]. Altered HRV has been associated with various urological conditions, including LUTS, neurogenic lower urinary tract dysfunction and overactive bladder [12,13,14]. Investigating HRV changes following TURP may provide insights into the broader physiological impact of the procedure, potentially linking urological recovery with autonomic stabilization.

Despite growing interest in the autonomic dimension of BPH, several gaps remain. First, few studies have integrated HRV analysis with validated symptom scores like the International Prostate Symptom Score (IPSS) to assess the physiological impact of TURP. Second, the temporal dynamics of autonomic recovery post-TURP are poorly understood, with most studies limited to short-term follow-up. Third, the correlation between HRV and specific symptom domains—such as voiding versus storage—has not been thoroughly investigated. Therefore, we conducted the first study to clarify the changes in autonomic nervous system function—specifically HRV—and voiding outcomes in patients with BPH following TURP. By integrating physiological and symptomatic assessments, this research aims to provide a more comprehensive understanding of TURP’s therapeutic impact beyond mechanical relief.

## 2. Materials and Methods

### 2.1. Study Design

This study was designed as a prospective, observational cohort investigation aimed at evaluating both autonomic nervous system (ANS) modulation and symptomatic outcomes in patients with benign prostatic hyperplasia (BPH) undergoing transurethral resection of the prostate (TURP). The primary endpoints included changes in heart rate variability (HRV), sympathovagal balance (quantified by low-frequency/high-frequency [LF/HF] ratio), and lower urinary tract symptoms (LUTS), assessed via validated clinical instruments. The study adhered to the STROBE (Strengthening the Reporting of Observational Studies in Epidemiology) guidelines to ensure methodological rigor and transparency. The study protocol was approved by the Institutional Review Board (IRB) of the tertiary medical center, and the investigation was conducted in accordance with the ethical principles of the Declaration of Helsinki.

### 2.2. Participants

A total of 242 male patients with clinically and radiologically confirmed BPH and bladder outlet obstruction (BOO) were prospectively enrolled between July 2018 and June 2024 at a tertiary medical center. Patients were assessed preoperatively and at a 3-month follow-up post-TURP for HRV, IPSS, and quality of life (QoL) metrics. Inclusion criteria were:

Age ≥ 50 years
Moderate to severe LUTS (IPSS ≥ 8).
Indication for TURP based on urodynamic or imaging evidence of BOO.
Ability to provide informed consent and comply with follow-up protocols.

Exclusion criteria included:

History of cardiovascular, neurological, or endocrine disorders known to affect autonomic function.
Use of medications influencing ANS activity (e.g., beta-blockers, anticholinergics).
Prior prostate surgery or pelvic radiation.
Incomplete baseline or follow-up data.

All participants discontinued 5–α reductase and α–blocker 2 weeks before surgery and remained off these medications until the 3-month postoperative visit. They should provide written informed consent before enrolling in this study. Of the initially enrolled 242 patients, all completed the study protocol without dropouts or exclusions for missing data, ensuring a complete dataset for analysis.

### 2.3. Data Collection Methods

To minimize confounding effects from daily activities and seasonal variation, Holter ECG recordings were scheduled during morning hours (08:00–11:00) under standardized clinical conditions. Patients were instructed to abstain from caffeine, alcohol, and vigorous physical activity for at least 24 h prior to monitoring. Although no formal seasonal adjustment was applied, the recruitment period spanned six years (2018–2024), which helped distribute data across different seasons and reduce seasonal bias. Data were collected at two time points: preoperatively (baseline) and three months postoperatively. Autonomic function was assessed using 24 h Holter electrocardiographic monitoring. HRV was analyzed using both time-domain (e.g., SDNN, RMSSD) and frequency-domain methods, with LF/HF ratio calculated to reflect sympathovagal balance. Data were processed using Kubios HRV Premium software (ver.3.0) under standardized conditions.

LUTS severity was evaluated using the International Prostate Symptom Score (IPSS), which includes seven items divided into storage (frequency, urgency, nocturia) and voiding (weak stream, intermittency, straining, incomplete emptying) domains. A separate QoL index was also recorded. All questionnaires were administered by trained personnel in a controlled clinical setting during morning hours to minimize circadian variability.

Additional clinical parameters such as age, body mass index (BMI), prostate volume (via transrectal ultrasound), and post-void residual urine volume were documented.

#### Data Analysis Techniques

Descriptive statistics were used to summarize demographic and clinical characteristics. Continuous variables were expressed as mean ± standard deviation (SD), and categorical variables as frequencies and percentages. Paired *t*-tests were employed to compare pre- and postoperative HRV metrics, LF/HF ratios, and IPSS. Subgroup analyses were conducted for IPSS storage and voiding domains.

Pearson correlation coefficients were calculated to assess the relationship between HRV parameters and voiding function scores. Multivariate linear regression models were constructed to adjust for potential confounders such as age, BMI, and prostate volume. Statistical significance was defined as *p* < 0.05. All analyses were performed using SPSS version 26.0 (IBM Corp., Armonk, NY, USA).

## 3. Results

### 3.1. Patient Characteristics

The cohort comprised 242 men with BPH (mean age: 67.4 ± 8.2 years, range: 52–82). Baseline prostate volume was 52.3 ± 15.7 mL, PSA was 3.8 ± 2.1 ng/mL, and PVR was 85.4 ± 45.2 mL. Most patients (59%, *n* = 143) were aged ≥65 years, and 53% (*n* = 127) had prostate volumes ≥ 50 mL. All underwent TURP without significant intraoperative complications.

#### Changes in HRV, LUTS, and QoL

**Figure 1:**
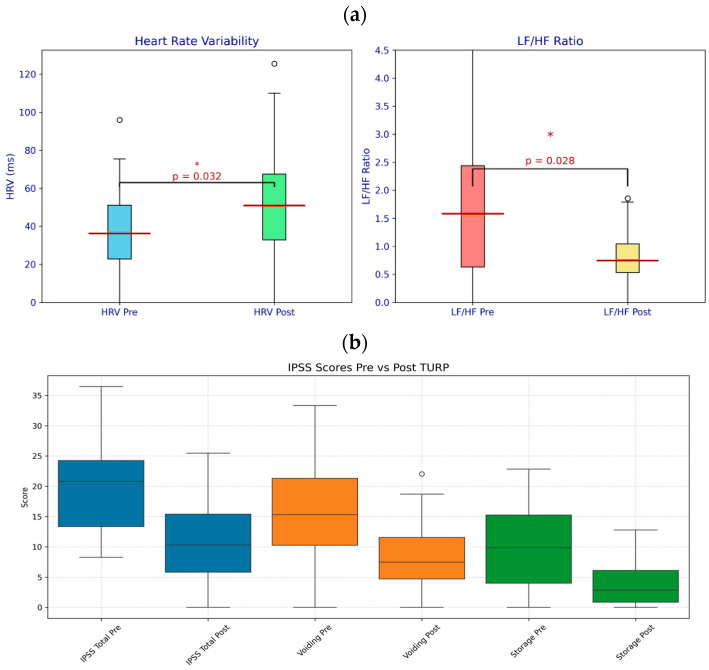
(**a**) HRV Pre vs. Post TURP: Boxplot showing HRV (SDNN in ms) before (blue, mean ~40 ms) and after (orange, mean ~60 ms) TURP, with a clear increase. Outliers are circles. LF/HF Ratio Pre vs. Post TURP: Boxplot of LF/HF ratio before (blue, mean ~2.5) and after (orange, mean ~0.5) TURP, showing a significant drop. Outliers are circles. (* means statistically significant results) (**b**) Boxplot of IPSS: Displays total (blue), voiding (orange), and storage (green) subscores before and after TURP, showing reductions. Outliers are circles.

Table 1 summarizes key outcomes. HRV (SDNN) increased from 36.97 ± 22.80 ms to 51.67 ± 27.59 ms (*p* = 0.032), indicating enhanced autonomic function (Figure 1a). The LF/HF ratio decreased from 1.63 ± 1.60 to 0.73 ± 0.52 (*p* = 0.028), suggesting improved sympathovagal balance with greater parasympathetic dominance (Figure 1a). Total IPSS decreased significantly from 18.5 ± 6.2 to 8.3 ± 4.1 (*p* < 0.001), reflecting reduced LUTS severity. Voiding subscores improved from 12.3 ± 4.5 to 5.2 ± 3.2 (*p* = 0.004), and storage subscores from 6.2 ± 2.1 to 3.1 ± 1.8 (*p* = 0.002), indicating relief of both obstructive and irritative symptoms (Figure 1b). QoL scores improved from 3.5 ± 1.2 to 1.8 ± 0.9 (*p* = 0.003), reflecting enhanced patient well-being.

### 3.2. Correlations

**Figure 2:**
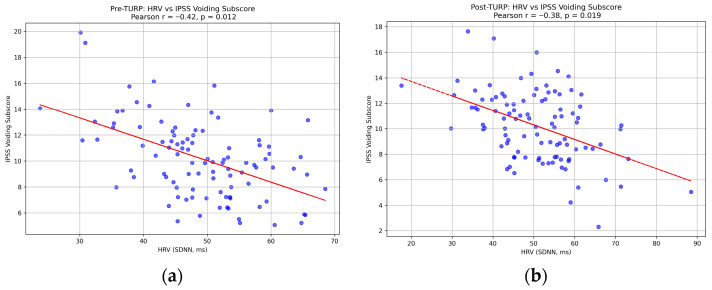
(**a**) Pre-TURP: Scatter plot with regression line showing inverse HRV-voiding score correlation (r = −0.42, *p* = 0.012). (**b**) Post-TURP: Scatter plot with regression line showing persistent correlation (r = −0.38, *p* = 0.019).

HRV (SDNN) showed a moderate inverse correlation with IPSS voiding subscore pre-TURP (r = −0.42, *p* = 0.012) (Figure 2a) and post-TURP (r = −0.38, *p* = 0.019) (Figure 2b), indicating that lower HRV was associated with more severe voiding symptoms. A weaker but significant correlation with the storage subscore (r = −0.29, *p* = 0.045) suggested autonomic influence on irritative symptoms. These correlations persisted after adjusting for age, BMI, and prostate volume, confirming HRV’s independent association with LUTS severity.

### 3.3. Subgroup Analysis

To explore the influence of baseline characteristics on outcomes, patients were stratified by age (<65 years vs. ≥65 years) and prostate volume (<50 mL vs. ≥50 mL). In patients aged ≥65 years (*n* = 143), the postoperative increase in HRV was more pronounced (ΔHRV: 18.2 ± 9.3 ms) compared to those <65 years (*n* = 99, ΔHRV: 11.4 ± 7.8 ms, *p* = 0.041). Similarly, patients with larger prostate volumes (≥50 mL, *n* = 127) exhibited greater improvement in the IPSS voiding subscore (Δvoiding subscore: 7.8 ± 3.5) compared to those with smaller prostates (<50 mL, *n* = 115, Δvoiding subscore: 5.9 ± 2.8, *p* = 0.033). These findings suggest that older patients and those with larger prostates may derive greater autonomic and symptomatic benefits from TURP, potentially due to more severe baseline obstruction and autonomic dysregulation.

#### Safety and Adverse Events

No significant perioperative complications, such as severe bleeding or infection, occurred. Four patients (1.65%) experienced post-TURP bleeding requiring repeated endoscopic hemostasis. Ten patients (4.16%) developed transient urinary incontinence, which resolved within 6 weeks post-TURP. No new-onset cardiac arrhythmias or other ANS-related adverse events were observed during the follow-up period, supporting the safety of TURP in this cohort.

## 4. Discussion

To our knowledge, this is the first study to correlate HRV with specific LUTS domains (voiding and storage) before and after TURP, highlighting HRV’s potential as a biomarker for BPH management. The findings also underscore the multifaceted therapeutic impact of TURP, demonstrating significant improvements in both LUTS and ANS function. The observed increase in HRV from 36.97 ± 22.80 ms to 51.67 ± 27.59 ms (*p* = 0.032) and the reduction in the LF/HF ratio from 1.63 ± 1.60 to 0.73 ± 0.52 (*p* = 0.028) indicate enhanced parasympathetic activity and improved sympathovagal balance after TURP. These autonomic changes, combined with substantial reductions in the IPSS total score (from 18.5 ± 6.2 to 8.3 ± 4.1, *p* < 0.001), voiding subscore (from 12.3 ± 4.5 to 5.2 ± 3.2, *p* = 0.004), storage subscore (from 6.2 ± 2.1 to 3.1 ± 1.8, *p* = 0.002), and QoL score (from 3.5 ± 1.2 to 1.8 ± 0.9, *p* = 0.003), reinforce TURP’s efficacy in alleviating BPH-related symptoms while promoting systemic physiological recovery.

The improvement in HRV suggests that TURP mitigates the chronic sympathetic overactivity often associated with BPH-induced bladder outlet obstruction. This finding aligns with previous research by McVary et al. [9], who reported autonomic nervous system overactivity in men with LUTS secondary to BPH, characterized by elevated sympathetic tone and reduced HRV. The observed shift toward parasympathetic dominance post-TURP may be attributed to reduced bladder outlet resistance, which alleviates the chronic stress on the detrusor muscle and pelvic neural pathways. This is further supported by Juszczak et al. [12], who noted that LUTS in BPH patients can exacerbate autonomic dysfunction through heightened sympathetic stimulation. The reduction in the LF/HF ratio in our study indicates a rebalancing of autonomic tone, which may have broader implications for cardiovascular health, as higher HRV is associated with reduced risk of cardiovascular events, such as arrhythmias or myocardial infarction [15,16], though direct evidence in BPH populations is lacking. This is particularly relevant given Fiev et al.’s findings of compromised autonomic profiles in LUTS patients with ischemic heart disease, suggesting that BPH-related autonomic dysfunction may exacerbate cardiovascular risk [17]. Related autonomic enhancements have been reported with noninvasive electromagnetic modalities (e.g., PAPIMI), which improved HRV and pain perception in chronic musculoskeletal patients [18].

Our findings reaffirm TURP as an effective and safe intervention for managing BPH with BOO, with significant and clinically meaningful improvements in IPSS and QoL. The substantial reduction in IPSS and QoL indices post-TURP, coupled with minimal adverse events (e.g., 1.65% post-TURP bleeding, 4.16% transient incontinence resolving within 6 weeks), underscores its safety profile and clinical utility. This aligns with recent evidence from a systemic review and meta-analysis by Porto et al. (2024), which demonstrated that TURP effectively relieves BOO in BPH patients, leading to sustained improvements in voiding function and quality of life over a 12-month follow-up period, with a low complication rate [19]. These outcomes highlight TURP’s role as a gold-standard treatment, offering both symptomatic relief and enhanced well-being, particularly when tailored to patient-specific factors such as age and prostate size.

The moderate inverse correlation between HRV and the IPSS voiding subscore both preoperatively (r = −0.42, *p* = 0.012) and postoperatively (r = −0.38, *p* = 0.019) indicates that diminished HRV (i.e., reduced parasympathetic tone) is associated with more severe voiding symptoms such as weak stream, straining, and incomplete emptying. This relationship persisted after TURP, suggesting that while surgical intervention alleviates mechanical obstruction, underlying autonomic imbalances may contribute to residual voiding dysfunction [20]. These observations align with evidence from case–control studies showing unfavorable cardiac autonomic profiles, including lower very low-frequency HRV components, in men with LUTS compared to asymptomatic controls [9,12,14]. Furthermore, the weaker but significant inverse correlation with the IPSS storage subscore (r = −0.29, *p* = 0.045) implies a nuanced ANS involvement in irritative symptoms, possibly driven by sympathetic overactivity, as noted in systematic reviews evaluating ANS dysregulation in LUTS associated with BPH [14,16].

Subgroup analyses also elucidate how baseline characteristics influence outcomes. Older patients (≥65 years) demonstrated a more substantial postoperative HRV increase (ΔHRV: 18.2 ± 9.3 ms) than younger counterparts (ΔHRV: 11.4 ± 7.8 ms, *p* = 0.041), likely due to age-related parasympathetic decline exacerbating baseline dysregulation. Similarly, those with larger prostate volumes (≥50 mL) experienced greater voiding subscore improvements (Δvoiding subscore: 7.8 ± 3.5 vs. 5.9 ± 2.8, *p* = 0.033), possibly reflecting more severe obstructive and autonomic burdens at baseline. The present study on TURP in BPH patients echoes findings from Shim et al. [21], who examined alpha-blocker (alfuzosin) effects on LUTS. Both demonstrate treatment-induced autonomic restoration: TURP enhanced HRV and reduced the LF/HF ratio, reflecting parasympathetic recovery, while alfuzosin converged LF/HF ratios from hypo- (0.89) and hyperactive (3.93) baselines to ~1.79, indicating balanced ANS activity. Similarly, both reported significant IPSS and Qmax improvements, supporting ANS dysregulation in LUTS pathophysiology. However, TURP showed persistent HRV-IPSS correlations postoperatively and greater benefits in older/larger-prostate subgroups, absent in Shim’s analysis, suggesting TURP’s broader systemic impact beyond pharmacological modulation. Older patients often present with advanced BPH and comorbidities that exacerbate autonomic imbalance, making the restoration of bladder outlet dynamics particularly impactful [22]. Similarly, larger prostate volumes are associated with greater mechanical obstruction, and their resection may lead to more significant symptom relief and autonomic recovery. These findings highlight the importance of tailoring TURP candidacy based on patient-specific factors, such as age and prostate size, to optimize outcomes [23,24].

Clinically, the ~40% increase in SDNN and ~55% decrease in LF/HF ratio surpass thresholds for meaningful autonomic recovery [15], potentially translating to reduced LUTS burden (via mitigated sympathetic drive on bladder function) and lower cardiovascular risk (e.g., fewer arrhythmias) [17,25]. Nonetheless, these changes’ prognostic value for hard endpoints like myocardial infarction or LUTS recurrence warrants validation in larger, longitudinal cohorts.

Collectively, these results support HRV as a valuable non-invasive biomarker for gauging LUTS severity, predicting treatment response, and monitoring autonomic recovery in BPH. Integrating HRV assessments into clinical practice could enhance risk stratification and guide treatment decisions, particularly for patients with comorbidities. For instance, combining HRV with biomarkers like Prostate-Specific Antigen (PSA) could refine personalized BPH management, optimizing outcomes for high-risk subgroups [25]. Despite its valuable insights, this study has several limitations. First, the observational design precludes definitive causal inference regarding the relationship between TURP, autonomic function, and LUTS improvement. While significant changes were observed, the underlying mechanisms—such as neural pathway modulation or inflammatory marker reduction—remain speculative. Second, the absence of a control group, such as BPH patients managed with medical therapy or watchful waiting, precludes definitive attribution of observed improvements solely to TURP, as natural history or placebo effects could contribute. Third, the 3-month follow-up period is relatively short, leaving the long-term durability of autonomic and symptomatic improvements uncertain. Fourth, potential confounders such as seasonal variation and lifestyle factors (e.g., caffeine intake, physical activity, sleep quality) may have influenced HRV measurements. While the study protocol included standardized morning monitoring and pre-test behavioral instructions, no formal seasonal stratification or activity logging was implemented. Finally, reliance on subjective IPSS and QoL scores introduces potential reporting bias, despite their validated clinical utility.

To address these limitations, future randomized controlled trials (RCTs) incorporating active comparators (e.g., alpha-blockers, 5-alpha-reductase inhibitors, or minimally invasive procedures) are essential to isolate TURP’s causal impact on autonomic function and symptomatic relief. Longitudinal studies with extended follow-up periods (e.g., 1–5 years) are needed to assess the sustainability of autonomic improvements and their impact on cardiovascular outcomes. Incorporating additional HRV metrics, such as frequency-domain measures (e.g., root mean square of successive differences, RMSSD) and time-domain analyses, could provide a more comprehensive assessment of autonomic function [26].

## 5. Conclusions

To our knowledge, this is the first study to correlate HRV with specific LUTS domains before and after TURP, highlighting HRV’s potential as a biomarker for BPH management. TURP significantly improves LUTS, QoL, and ANS function. Inverse correlations between HRV and IPSS voiding and storage subscores support HRV’s utility in assessing LUTS severity and treatment response. Greater benefits in older patients (≥65 years) and those with larger prostates (≥50 mL) underscore TURP’s efficacy in high-risk groups. Longitudinal studies are needed to validate HRV’s prognostic role and to clarify long-term cardiovascular outcomes, paving the way for integrated urological and systemic health management.

## Figures and Tables

**Table 1:** Pre- and Post-TURP Changes in HRV, IPSS, and QoL.

Parameter	Pre-TURP (Mean ± SD)	Post-TURP (Mean ± SD)	Mean Difference (Δ)	95% CI	SE	*p*-Value	Power
HRV (SDNN, ms)	36.97 ± 22.80	51.67 ± 27.59	+14.70	2.1 to 27.3	6.30	0.032	0.81
LF/HF Ratio	1.63 ± 1.60	0.73 ± 0.52	−0.90	−1.6 to −0.2	0.42	0.028	0.78
Total IPSS	18.5 ± 6.2	8.3 ± 4.1	−10.2	−12.1 to −8.3	1.02	<0.001	>0.99
Voiding Subscore	12.3 ± 4.5	5.2 ± 3.2	−7.1	−8.4 to −5.8	0.65	0.004	0.93
Storage Subscore	6.2 ± 2.1	3.1 ± 1.8	−3.1	−3.7 to −2.5	0.31	0.002	0.91
QoL Score	3.5 ± 1.2	1.8 ± 0.9	−1.7	−2.1 to −1.3	0.20	0.003	0.89

Note: Mean differences (Δ), 95% confidence intervals (CI), standard errors (SE), and post hoc power estimates were calculated for each comparison.

## Data Availability

The data presented in this study are available on request from the corresponding author with a reasonable reason.

## Abbreviations

The following abbreviations are used in this manuscript

BPH	Benign Prostate Hyperplasia
LUTS	Lower Urinary Tract Symptoms
QoL	Quality of Life
TURP	Transurethral Resection of Prostate
BOO	Bladder Outlet Obstruction
ANS	Autonomic Nervous System
HRV	Heart Rate Variability
IPSS	International Prostate Symptom Score
SDNN	Standard Deviation of normal-to-Normal Intervals
L/F/HF	Lower Frequency/High Frequency
BMI	Body Mass Index
PSA	Prostate Specific Antigen
